# Transverse Magnetoresistance Induced by the Nonuniformity of Superconductor

**DOI:** 10.3390/nano12081313

**Published:** 2022-04-12

**Authors:** Duo Zhao, Zhiyuan Zhao, Yaohan Xu, Shucheng Tong, Jun Lu, Dahai Wei

**Affiliations:** 1 State Key Laboratory of Superlattices and Microstructures, Institute of Semiconductors, Chinese Academy of Sciences, Beijing 100083, China; duozhao@semi.ac.cn (D.Z.); zyzhao@semi.ac.cn (Z.Z.); yhxu@semi.ac.cn (Y.X.); 2 Center of Materials Science and Optoelectronics Engineering, University of Chinese Academy of Sciences, Beijing 100190, China; 3 Beijing Academy of Quantum Information Sciences, Beijing 100193, China; tongsc@baqis.ac.cn (S.T.); lujun@semi.ac.cn (J.L.)

**Keywords:** transverse magnetoresistance, nonuniform superconductivity, vortices, separation

## Abstract

The transverse magnetoresistance (*R_xy_*) caused by inhomogeneous superconductivity is symmetric about the magnetic field around the critical magnetic field region. This has caused many disturbances during the study of vortex dynamics by Hall signals. Here, we found that the peak of *R_xy_* measured in our samples was induced by the nonuniformity of the superconductors. The peak values of *R_xy_* decrease with increasing applied current and temperature, which can be described by the theory of superconductivity inhomogeneity. Based on this, we have proposed and verified a method for separating the transverse voltage caused by the inhomogeneity of superconductivity. Additionally, quantity Δ*B*(0) can also be used to characterize the uniformity of superconductivity. This clears up the obstacles for studying vortex motion dynamics and reveals a way to study the influence of the domain wall on superconductivity.

## 1. Introduction

Hall signals reflecting abundant physical effects in magnetic materials and semiconductors can also be used to study the transport behavior of vortices in superconductors [1,2,3]. In general, there are two types of transverse magnetoresistance signals in superconductors: even-in-field transverse voltage (ETV) and odd-in-field transverse voltage (OTV) [4]. In type II superconductors, when the symmetry of vortex movement is broken, so-called guiding motion vortices would appear, which would induce the ETV. The appearance of OTV is caused by the Magnus force of the vortex, under a magnetic field. These two kinds of Hall signals have potential applications for transporting spin information and revealing the mechanism of high-temperature superconductors by Nernst signals [5,6,7,8,9,10,11].

ETV has been widely used to characterize vortex motion. The influence of magnetic domain walls on the movement of the vortex has been widely studied through ETV [10,11,12]. In addition, vortex guiding motion would also be generated by the crystal structure with an anisotropic pinning effect, such as the twin boundary interface [13] and artificially generated anisotropic pinning geometries (lithographic patterning) [14,15,16,17,18]. However, even in isotropic superconductor films, ETVs have also been reported with random emergence. The inhomogeneity of superconductors would also cause the appearance of ETV [19,20]. Furthermore, even very small superconducting inhomogeneities can also lead to ETV. Then, EVT induced by this mechanism would cause disturbance and it is difficult to analyze vortex dynamics using the Hall effect. It also is a very meaningful subject to separate the ETV signal caused by vortex motion from the inhomogeneity of superconductivity [21,22]. In general, the inhomogeneity of superconductors could be induced by nonuniform superconducting film [20] or the complex magnetic structure in superconducting/ferromagnet (S/F) heterostructure [19]. Moreover, S/F heterostructures rich in novel physical effects such as spin-triplet Cooper pairs [23,24,25,26,27,28] and Majorana zero-energy mode in skyrmion/superconducting heterostructures have received considerable attention [29,30,31,32,33,34]. ETV with magnetized history is the most typical feature as reported by J. E. Villegas et al. [19] in S/F heterostructure.

In this paper, we would make further research on EVT caused by inhomogeneity of the superconductivity based on the research result before [19,20]. Additionally, Nb film was used as the superconductor layer, and the correlation between ETV and *R_xx_* caused by the inhomogeneity of superconductivity was studied under an external magnetic field using samples Nb (30 nm)/Co (5 nm) and Nb (30 nm). By analyzing the influence of different applied currents and temperatures on the ETV, it was found that the ETV generated by vortex motion [13,35] and the inhomogeneity of superconductivity have the opposite behavior. At the same time, we established the correlation of the ETVs when the magnetic field was parallel and perpendicular to the sample plane, which provided an effective way to separate the ETV caused by the inhomogeneity of the superconductor from vortex guiding motion. The quantity Δ*B*(0) can also be used to characterize the uniformity of superconductivity.

## 2. Experiment

**Figure 1:**
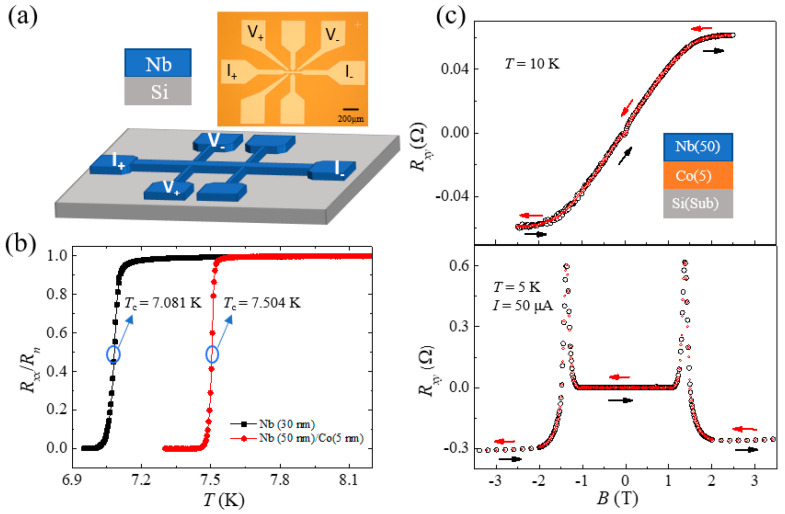
(**a**) Sketch structures and the optical microscopy of a typical Hall bar device, with a standard four-terminal configuration for measuring the transverse resistance. (**b**) The *R*_N_-*T* curve of samples Nb (30 nm) black dots and Nb (50 nm)/Co (5 nm) red dots, and *R*_N_ = *R_xx_*/*R_n_*. (**c**) The *R_xy_* as a function of the applied magnetic field *B* of the Nb (50 nm)/Co (5 nm) at *T* = 10 K (top panel) and 5 K (bottom panel), respectively. Red and black arrows indicate the sweeping magnetic field direction.

The Co (5 nm)/Nb (50 nm) and Nb (30 nm) samples were grown on Si substrates by magnetron sputtering (base pressure 9 × 10^−6^ Pa). The thickness of the film can be controlled by the sputtering time according to the calibrated growth rate. A standard Hall device, as shown in Figure 1a, was fabricated with a 20 μm wide and 200 μm long channel, and the distance between the nearest probes along the current direction was 60 μm. When the temperature is lower than the critical temperature of the superconductor (*T*_C_ = 7.504 K for Co (5 nm)/Nb (50 nm) and 7.081 K for Nb (30 nm), as shown by Figure 1b), the *R_xx_* drops rapidly to zero. Hall bar devices are used to measure the ETV and longitudinal resistance *R_xx_* by four-terminal measurements in physical property measurement system (PPMS). *T*_C_ is the temperature corresponding to *R* = *R_n_*/2 and *R_n_* is the normal state resistance, as shown in Figure 1b. *R_xx_* and *R_xy_* are measured at various temperatures and the applied currents. The accuracy of the measured temperature is ±1 mK. Additionally, the accuracy of measured magnetic field *B* is more than 10^−5^ T.

## 3. Results and Discussion

**Figure 2:**
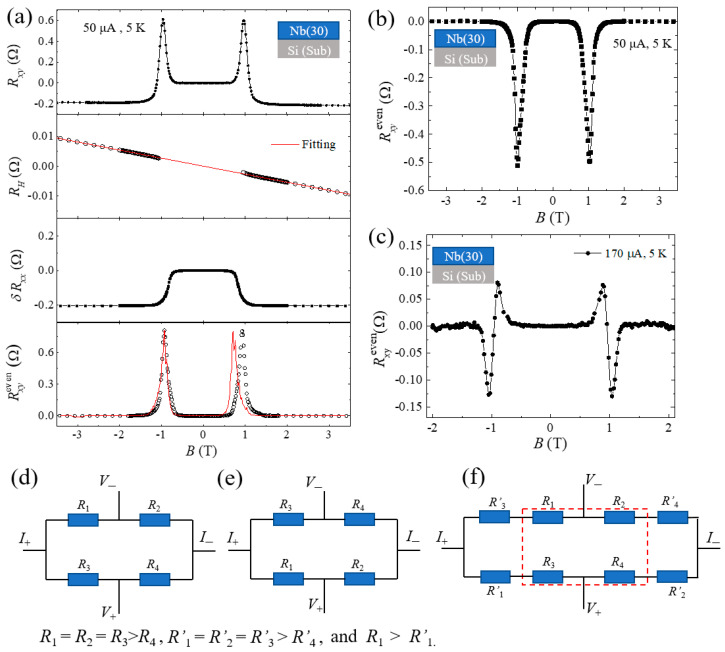
(**a**) The *R_xy_*, δ*R*_H_, *R_xx_* and $R_{xy}^{even}$ changing with *B* from the top-to-bottom panel; the red line of the bottom panel is calculated by the four-resistance method. (**b**,**c**) are $R_{xy}^{even}$-*B* curve with opposite polarities shapes of peaks and oscillating behavior in other devices, respectively. (**d**–**f**) are the schematic structure of the four-resistance model.

During sweeping field *B* (from –3.5 to 3.5 T) at *T* = 10 K above the *T*_C_, the *R_xy_*-*B* curve was shown by the top panel of Figure 1c. The contribution of anomalous Hall effect (AHE) in the Co layer can be easily distinguished with in-plane magnetic anisotropy. However, when the temperature is below *T*_C_, i.e., *T* = 5 K, *R_xy_* is distinct and much more complex, as shown by Figure 1c bottom panel and Figure 2a top panel for Co/Nb and Nb, respectively. Under field cycling, *R_xy_* is without hysteresis. The anomalous *R_xy_* peaks appear at approximately *B* = ±1.3 T for Co/Nb and around *B* = ±1.03 T for Nb, which are close to the critical magnetic field *B*_C_ (field *B* corresponding to *R* = *R_n_*/2).

In a general case of the S/F bilayer, the transverse resistance can be expressed as,

(1) $$R_{xy}=\frac{V_{xy}}{I_{0}}=\left(R_{H}+R_{\mathrm{AHE}}+\delta R_{xx}+R_{xy}^{even}\right)$$

where *R*_H_ and $R_{\mathrm{AHE}}$ are the ordinary and anomalous Hall resistance, respectively. They would have zero contribution in the superconducting state since no current flows in the Co layer. $\delta R_{xx}$ is the longitudinal component mixed with the transverse channel due to the imperfection of the Hall device, with a small factor of δ = 1.6% for our device. $R_{xy}^{even}$ is the abnormal even transverse resistance appearing near the critical magnetic field and would be discussed in detail next.

The typical results for Nb (30 nm) are shown in Figure 2a. And measured *R_xy_* is plotted on the top panel. According to Equation (1), the *R_xy_* can be separated into the *R*_H_, mixed longitudinal resistance and the abnormal even resistance, where the AHE is absent since the Co layer is removed. As shown in the second panel, the Hall resistance has a linear relation of *R*_H_ = *kB* in the normal states for *B* above the critical fields, where *k* = –0.0027 Ω/T. The mixed component $\delta R_{xx}$ is around −0.203 Ω as shown in the third panel. Both *R*_H_ and $\delta R_{xx}$ drop to zero within the superconductive state. Thus the $R_{xy}^{even}$ can be extracted, with two distinct peaks at *B* = ±1.03 T as plotted in the bottom panel of Figure 2a. $R_{xy}^{even}$ of the S/F can also be extracted by a proper subtraction of the AHE resistance and are similar to that of single S layer. In the case of the relatively thick (5 nm) Co layer deposited on Nb, its magnetic anisotropy is in-plane, which is dominated by the demagnetization field. The anomalous Hall effect (*R_xy_*) is shown by Figure 1c top panel. The black and red symbols represent the *B* field sweeps up and down, respectively. The *R_xy_*-*B* loops do not show any hysteresis, indicating the out-of-plane as the magnetic hard axis. The zero remanence at *B* = 0, confirms that it would turn to demagnetized states when the applied perpendicular field is removed, and there should not be a memory effect in such a hard axis. Therefore, the transverse magnetoresistance in Nb/Co heterostructure as shown by Figure 1c bottom panel, does not show any hysteresis and field cycling effects. The black and red circles for the magnetic field sweeping up and down are indistinguishable. Such results are quite different with that of ref. [19], in which the magnetic nanodots have clear hysteresis with the magnetic vortex states. Then, we only display and discuss the results of a single S layer next.

*R_xy_* and the extraction of $R_{xy}^{even}$ in different devices are reproducible for a given device, while the $R_{xy}^{even}$ varies among devices. We have observed different polarity shapes of peaks, as shown in Figure 2b,c for another two devices with single Nb (30 nm). In all cases, the abnormal peaks are always appearing at around *B*_C_ and even-symmetry about magnetic field. The random polarity and shapes of the abnormal peaks in $R_{xy}^{even}$ strongly suggest that they could correlated to the inhomogeneity in the system. The Nb layers deposited at room temperature by magnetron sputtering are amorphous, and the motion of the vortices is isotropic. There are also considerable amounts of defects. Furthermore, when an in-plane magnetic field is applied, $R_{xy}^{even}$ still exists and its peak value is not significantly reduced (this would be displayed in detail next). The contribution of the vortex motion was ruled out. Therefore, $R_{xy}^{even}$ would be related to the inhomogeneity of superconductivity in the Nb layer. A. Segal et al. developed a method to interpret $R_{xy}^{even}$ using *R_xx_*, as shown in Figure 2d, see ref. [20] in detail. According to the four-resistor network model, the correlation between $R_{xy}^{even}$ and *R_xx_* is finally expressed as

(2) $$R_{xy}^{even}=\frac{\Delta T}{4}\frac{\partial R_{xx}\left(T,B\right)}{\partial T}+\frac{\Delta B}{4}\frac{\partial R_{xx}\left(T,\;B\right)}{\partial B}$$

where Δ*T* and Δ*B* represent the difference in *T*_C_ and *B*_C_ of the inhomogeneous superconductors. Δ*B* can also be used to describe the nonuniformity of superconductivity.

In our case, the temperature was kept at *T* = 5 K during sweeping the field, and the first term $\frac{\partial R_{xx}\left(T,B\right)}{\partial T}=0$. Thus, we focus on the second term, i.e., the partial derivatives of *R_xx_* to magnetic field *B* and use $\frac{\Delta B}{4}$ for the correction peak value. The red solid line shown at the bottom panel of Figure 2a was fit based on Equation (2), which describes the change of $R_{xy}^{even}$ (black circles) with *B*. The values of Δ*B* can reflect the unevenness of superconductivity, and the larger value of Δ*B* is, the more uneven the superconductivity. This model perfectly explains the mechanism causing *R*_ETV_ in our experiment. and negative peak value of $R_{xy}^{even}$ ($R_{xy}^{p}$ = −0.51 Ω) also appeared in other devices, as displayed in Figure 2b. The negative and positive peak value of $R_{xy}^{even}$ is a random appearance in different Hall devices. The negative $R_{xy}^{even}$ can also be interpreted by this model, as shown in Figure 2e. The random distribution of the nonuniform superconductivity causes the random distribution of the resistances. Based on the four-resistance models, we assume the simplest case, i.e., that the distribution of the resistance is as shown in Figure 2e. This distribution of the resistances *R*_1_, *R*_2_, *R*_3_, *R*_4_ are antisymmetric about current direction with the resistances shown in Figure 2d. Obviously, $R_{xy}^{even}$ measured through the model shown in Figure 2e should be negative, if the value of the $R_{xy}^{even}$ measured through the model shown in Figure 2d is positive. In addition, the oscillation of $R_{xy}^{even}$ around *B*_C_ is also observed, as shown in Figure 2c. This was caused by multiple uneven regions of superconductivity and can be regarded as the result of $R_{xy}^{even}$ shown in Figure 2a’s bottom panel plus $R_{xy}^{even}$ shown in Figure 2b, and the *B*_C_ of these two $R_{xy}^{even}$, are not equal. As shown in Figure 2f, it can be regarded as a series circuit shown in Figure 2d,e, and the distribution of the resistors circled in red box and the out of side are the same with the circuit shown in Figure 2d and Figure 2e, respectively. The condition of *R*_1_ > *R*’_1_ guarantees that the *B*_C_ of the two circuits is different.

**Figure 3:**
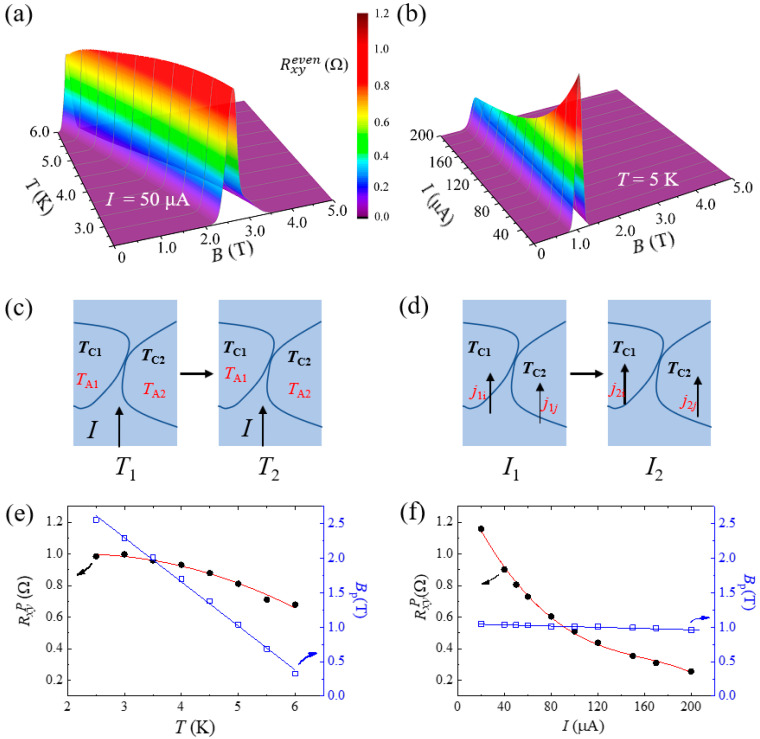
(**a**,**b**) show the changes in $R_{xy}^{even}$ with temperature and applied current, respectively. A color scale bar is shown in the middle. (**c**,**d**) are the sketched maps of an inhomogeneous superconductor during current flowing through the Hall bar under different temperature and applied currents. The arrows represent the direction of the applied currents. (**e**,**f**) Peak values of $R_{xy}^{even}$ (black dots) and the corresponding fields (blue squares) as a function of temperatures and current densities, respectively. The red lines are the theoretical fitting.

However, the ETV produced by the inhomogeneity of superconductors would likely cause disturbances when studying vortices motion. Distinguishing and separating these two voltages are crucial for the proper interpretation of the vortices. Based on this, we systematically studied $R_{xy}^{even}$ at different temperatures and current densities in Nb (30 nm) single-layer film. $R_{xy}^{even}$ changes with *B* at different temperatures are obtained with applied current *I* = 50 μA, as shown in Figure 3a. The magnetic field *B*_p_ values corresponding to peak ($R_{xy}^{p}$) decrease from 2.55 T to 0.33 T with temperature (*T*) increasing from 2.5 K to 6 K as shown by the blue squares in Figure 3e. It can be fit well with Ginzburg–Landau theory (blue lines): *B*_p_(*T*) = *B*_p_(0)(1 − *T*/*T_C_*^0^). The *B*_p_-*T* curve is consistent with *B_c_*^⊥^ (*T*), where *B_c_*^⊥^ is critical field applied to out of the plane. $R_{xy}^{p}$ decreases from approximately 1 Ω to 0.67 Ω as the temperature increases. To quantitatively describe this trend, $R_{xy}^{p}$ changing with *T* is shown by black dots in Figure 3e. According to Equation (2), it is proportional to Δ*B*. Therefore, the changing trend shown in Figure 3e can be regarded as the influence of temperature on Δ*B*. Assuming that *B*_1_ and *B*_2_ are the lowest and highest critical magnetic field values in the inhomogeneous superconductor, respectively, and the critical temperatures are *T*_C1_ and *T*_C2_, respectively, then Δ*B = B*_2_ *− B*_1_. As shown in Figure 3c, the regions of *T*_C1_ and *T*_C2_ are the distribution of the inhomogeneous superconductivity. The current densities are different due to the distinction of the critical magnetic field between the two regions. Therefore, the temperatures are also different for the Joule heating of the current flowing through these two regions. With the temperature increasing from *T*_1_ to *T*_2_, the value of Δ*B* would be changed. According to the relationship of Corter–Casimir’s two-fluid model,

(3) $$\Delta B\left(T\right)=B_{2}\left(T\right)-B_{1}\left(T\right)=B_{2}\left(0\right)\left[1-{\left(\frac{T+T_{A2}}{T_{C2}}\right)}^{2}\right]-B_{1}\left(0\right)\left[1-{\left(\frac{T+T_{A1}}{T_{C1}}\right)}^{2}\right]$$

where *T_A_*_1_ and *T_A_*_2_ represent the equivalent temperature with the Joule heating of the current flowing through the nonuniform superconducting regions as shown by Figure 3c. The correlation of ∆*B* with *T* can be expressed as,

(4) $$\Delta B\left(T\right)=aT^{2}+bT+c$$

where $a=\frac{B_{1}\left(0\right)}{T_{c1}{}^{2}}-\frac{B_{2}\left(0\right)}{T_{c2}{}^{2}}$, $b=\frac{2T_{A1}B_{1}\left(0\right)}{T_{c1}{}^{2}}-\frac{2T_{A2}B_{2}\left(0\right)}{T_{c2}{}^{2}}$ and $c=B_{2}\left(0\right)-B_{1}\left(0\right)+\frac{B_{1}\left(0\right)T_{A1}}{T_{c1}{}^{2}}-\frac{B_{2}\left(0\right)T_{A2}}{T_{c2}{}^{2}}$. Finally, the changes in $R_{xy}^{p}$ with T were better fitted by Equation (4), as shown by the red line in Figure 3e. This change feature is contrary to the motion of the vortices. It is obvious that the thermal excitation of the magnetic flux vortices overcomes the defect pinning effect with increasing temperature, and more vortices move to produce $R_{xy}^{even}$. Therefore, this trend can be used to distinguish these two kinds of mechanisms.

$R_{xy}^{p}$ can also be changed by the current density. We measured $R_{xy}^{even}$ by sweeping *B* with different applied currents at *T* = 5 K, as shown in Figure 3b. When the applied current changes from 20 to 200 μA, $R_{xy}^{p}$ decreases from 1.15 Ω to 0.25 Ω. $R_{xy}^{p}$ changing with *T* is shown by black dots in Figure 3f. However, *B*_p_ (corresponding the peaks $R_{xy}^{p}$) slight decreases from 1.049 T to 0.958 T with the current increasing from 20 μA to 200 μA, as shown by the blue squares in Figure 3f. 

The influence of the current density on $R_{xy}^{even}$ can also be explained by that on Δ*B*. As shown in Figure 3d, when the current *I*_1_ flows through the non-uniform superconducting regions *T*_C1_ and *T*_C2_, the current densities *j*_1i_ and *j*_1j_ are different due to the difference of the critical magnetic field in these two regions. Additionally, two effects would influence the superconducting critical magnetic field. One is current induced by the Oersted magnetic field, and the other is the Joule heating. According to the Silsbee rule, the Oersted magnetic field generated by the current can be expressed as ΔB=fI, while the temperature increases due to Joule heat is $\Delta T=dI^{2}$, where *f* and *d* are the constants of proportionality. When increasing the current from *I*_1_ to *I*_2_, the current densities *j*_2i_ and *j*_2j_ would change the value of Δ*B*. Therefore, according to the Corter–Casimir’s two-fluid model,

(5) $$\begin{array}{c} B_{1}\left(I\right)=B_{1}\left(0\right)\left[1-{\left(\frac{T+d_{1}I^{2}}{T_{C1}}\right)}^{2}\right]-f_{1}I,\;\\ B_{2}\left(I\right)=B_{2}\left(0\right)\left[1-{\left(\frac{T+d_{2}I^{2}}{T_{C2}}\right)}^{2}\right]-f_{2}I. \end{array}$$

and the influence of current on ∆*B* can be expressed as,

(6) $$\Delta B\left(I\right)=B_{2}\left(I\right)-B_{1}\left(I\right)=AI^{4}+CI^{2}+DI+F$$

where $A=\frac{B_{1}\left(0\right)d_{1}^{2}}{T_{C1}^{2}}-\frac{B_{2}\left(0\right)d_{2}^{2}}{T_{C2}^{2}}$, $C=\frac{2TB_{1}\left(0\right)d_{1}}{T_{C1}^{2}}-\frac{2TB_{2}\left(0\right)d_{2}}{T_{C2}^{2}}$, $D=f_{1}-f_{2}$ and $F=B_{1}\left(0\right)-B_{2}\left(0\right)+\frac{B_{1}\left(0\right)T^{2}}{T_{C1}^{2}}-\frac{B_{2}\left(0\right)T^{2}}{T_{C2}^{2}}$. It was better fitted by Equation (6) as the red line in Figure 3d using these four parameters. This change feature is contrary to the motion of the vortex. The movement of the vortex in the superconductor first overcomes the magnetic flux pinning effect caused by impurities or grain boundaries. The main force that overcomes the pinning action is the Lorentz force: $\vec{F}=\vec{J}\times \vec{B}$, where $\vec{J}$ represents the current density and $\vec{B}$ represents the magnetic flux density. Obviously, the Lorentz force increases with current density. Then, there will be more vortices overcoming the pinning potential and performing flux flow. $R_{xy}^{even}$ would also increase with current density. According to the above description and analysis, we found that the trends of the $R_{xy}^{p}$ values with the current density and temperature are significantly different from those of the vortex motion. Therefore, the mechanism causing $R_{xy}^{even}$ can be distinguished by the peak values changing with temperature or current.

**Figure 4:**
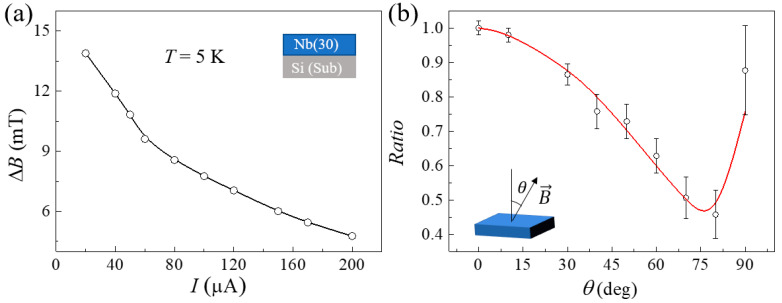
(**a**) Δ*B* as a function of current densities. (**b**) shows the *Ratio* = $\frac{P_{xx}^{\theta }}{P_{xx}^{⊥}}\frac{\Delta B^{\theta }}{\Delta B^{⊥}}$ (red line) and the experimental values *Ratio* = $\frac{R_{xy}^{p\theta }}{R_{xy}^{p⊥}}$ (black circles) changing with the angle. Insert is the definition of the angle θ.

As discussed above, $R_{xy}^{even}$ can be changed by current density and temperature. We attributed these to the change of Δ*B*. The contribution of $\frac{\partial R_{xx}\left(T,\;B\right)}{\partial B}$ was slight and neglected here. To confirm this, the change of Δ*B* with applied currents was displayed in Figure 4a. when the applied currents changed from 20 μA to 200 μA, Δ*B* decreased from 13.8 mT to 4.7 mT. the changing trend is similar to $R_{xy}^{even}$ as shown in Figure 3f. However, this may lead to that Δ*B* may not be a good quantity to characterize the uniformity of superconductivity. The uniformity of superconductivity is determined by the quantity of material itself, and it should not be affected by temperature and applied current. Therefore, we can get a quantity: Δ*B*(0)= *B*_2_(0) − *B*_1_(0) according to Equation (6) or (4). Δ*B*(0) would be a good quantity to characterize the uniformity of superconductivity.

To separate the $R_{xy}^{even}$ caused by a nonuniform superconductor from guide motion vortices, we studied the action of $R_{xy}^{even}$ caused by a nonuniform superconductor changing with *θ*. The definition of *θ* is shown by the insert of Figure 4b. According to the four-resistor network model,

(7) $$R_{xy}^{even}=\frac{\Delta B}{4}\frac{\partial R_{xx}\left(T,B\right)}{\partial B}$$

When the temperature is fixed, $R_{xy}^{even}$ is determined by Δ*B* and $\frac{\partial R_{xx}\left(T,B\right)}{\partial B}$, where $\frac{\partial R_{xx}\left(T,B\right)}{\partial B}$ can be obtained by the measured longitudinal resistance and $\Delta B=B_{c2}-B_{c1}$. where *B*_C1_ and *B*_C2_ represent the different maximum and minimum critical magnetic fields of the superconducting inhomogeneous region. $\frac{\Delta B^{⊥}}{\Delta B^{∥}}=\frac{B_{c}^{⊥}}{B_{c}^{∥}}$, where $\Delta B^{⊥}$ and $\Delta B^{∥}$ represent ΔB when the magnetic field is perpendicular and parallel to the sample plane, respectively. $B_{c}^{⊥}$ and $B_{c}^{∥}$ represent the critical magnetic field when the magnetic field is perpendicular and parallel to the sample plane, respectively. Therefore, from formula (2), it can be seen that the relationship between $R_{xy}^{even⊥}$ (perpendicular to the magnetic field) and $R_{xy}^{even∥}$ (parallel to the magnetic field) should be

(8) $$R_{xy}^{even⊥}=\frac{\frac{\partial R_{xx}^{⊥}}{\partial B}\;\Delta B^{⊥}}{\frac{\partial R_{xx}^{∥}}{\partial B}\;\Delta B^{∥}}R_{xy}^{even∥}$$

According to Equation (5), $R_{xy}^{p⊥}$ the peak value of $R_{xy}^{even⊥}$ can be obtained, and $\Delta B^{⊥}$ can also be obtained as $\Delta B^{⊥}=\frac{B_{c}^{⊥}}{B_{c}^{∥}}\Delta B^{∥}$. Then, $R_{xy}^{even⊥}$ would be obtained. In this way, the process of deriving $R_{xy}^{even⊥}$ from $R_{xy}^{even∥}$ is realized to separate $R_{xy}^{even}$ from $R_{xy}$ caused by vortex motion. To verify the correctness of this method, we analyzed the peak values at different *θ*. We define the $R_{xy}^{even}$ peak value ratio as $Ratio=\frac{R_{xy}^{p\theta }}{R_{xy}^{p⊥}}$ (Figure 4b black circles) and the $\frac{\partial R_{xx}\left(T,B\right)}{\partial B}$ peak value ratio as $Ratio=\frac{P_{xx}^{\theta }}{P_{xx}^{⊥}}\;\frac{\Delta B^{\theta }}{\Delta B^{⊥}}$ (Figure 4b red line), where $P_{xx}^{\theta }$, $P_{xx}^{⊥}$ and $R_{xy}^{p\theta }$ represent the peak values of $\frac{\partial R_{xx}^{\theta }}{\partial B}$, $\frac{\partial R_{xx}^{⊥}}{\partial B}$ and $R_{xy}^{even}$(*θ*). The trend of these two ratios was basically fitted well. This also further verifies the reliability of formula (5). We also repeated different samples, including S/F, and these two ratios also overlapped. However, when the magnetic field is parallel to the plane (*θ* = 90°) in Figure 4b, these two ratios are slightly different. The possible reason is that the magnetic field taking one point every 50 Oe is too sparse, and the peak width of $R_{xy}^{even}$ is very small when the magnetic field is parallel to the plane. There may be a relatively large error when calculating $\frac{\partial R_{xx}^{∥}}{\partial B}$, which may result in the difference shown in Figure 4b. Using this method to separate $R_{xy}^{even}$ is a reliable method. This kind of transverse magnetoresistance is very common in superconductors. When studying vortex motion, we must pay attention to it.

## 4. Conclusions

In our experiment, $R_{xy}^{even}$ originating from the inhomogeneity of superconductivity can be described by the simple model of four-resistance method mentioned by A. Segal et al. [20]. The peak values of transverse magnetoresistance decreasing with increasing applied current, and temperature can be well described by our modified four-resistance method. Additionally, quantity Δ*B*(0) can also be used to characterize the uniformity of superconductivity. Furthermore, we propose a way to separate it from the Hall signal caused by guiding motion. This provides a reliable way to study vortex motion in superconductors using Hall voltage.

## Data Availability

The data presented in this study are available on request from the corresponding author.
